# Supplementary material for: Incidence, Risk Factors, and Consequences of Post-Traumatic Stress Disorder Symptoms in Survivors of COVID-19-Related ARDS
Source: Int J Environ Res Public Health. 2023 Apr 13;20(8):5504. doi: 10.3390/ijerph20085504 (PMC10138688; doi:10.3390/ijerph20085504)
Supplement: Supplementary file 1 [file ijerph-20-05504-s001.zip › ijerph-2119073-supplementary.pdf]

Supplementary Materials

Table S1. The five EQ-5D-5L health dimensions in patients without and with PTSD symptoms.

EQ-5D-5L DIMENSION	LEVEL	No PTSD (n 104)	PTSD (n 34)	p-value
ANXIETY AND DEPRESSION (%)	1	75 (72.1)	14 (41.2)	0.001
	2	19 (18.3)	9 (26.5)	
	3	9 (8.7)	7 (20.6)	
	4	0 (0)	3 (8.8)	
	5	1 (1.0)	1 (2.9)	
MOBILITY (%)	1	68 (65.4)	16 (47.1)	0.004
	2	25 (24)	5 (14.7)	
	3	7 (6.7)	7 (20.6)	
	4	4 (3.8)	6 (17.6)	
	5	0 (0)	0 (0)	
PAIN-DISCOMFORT (%)	1	58 (55.8)	19 (55.9)	0,188
	2	31 (29.8)	6 (17.6)	
	3	11 (10.6)	5 (14.7)	
	4	4 (3.8)	4 (11.8)	
	5	0 (0)	0 (0)	
SELF CARE (%)	1	87 (83.7)	25 (73.5)	0,081
	2	13 (12.5)	4 (11.8)	
	3	3 (2.9)	5 (14.7)	
	4	1 (1)	0 (0)	
	5	0 (0)	0 (0)	
USUAL ACTIVITIES	1	74 (71.2)	21 (61.8)	0,041
	2	19 (18.3)	3 (8.8)	
	3	8 (7.7)	6 (17.6)	
	4	3 (2.9)	4 (11.8)	
	5	0 (0)	0 (0)	

Abbreviations: PTSD, post-traumatic stress disorder.

Table S2. Comparison of SF-36 score in the eight health domain and in Physical and Mental Component Score. between patients without and with PTSD symptoms.

SF-36 DOMAIN	No PTSD (n 104)	PTSD (n 34)	P value
Emotional well-being	70.19 (16.11)	59.68 (16.18)	0.002
Energy - Fatigue	54.77 (17.07)	47.50 (15.97)	0.030
General health	56.34 (20.95)	35.15 (25.89)	< 0.001
Pain	80.09 (25.78)	70.15 (30.23)	0.062
Physical functioning	83.70 (20.36)	74.41 (26.79)	0.034
Role limitations due to physical health	60.88 (47.02)	48.53 (49.60)	0.190
Role limitations due to emotional problems	83.64 (34.56)	54.90 (48.47)	< 0.001
Social functioning	80.32 (26.32)	61.76 (37.68)	0.002
PCS (Physical Component Score)	44.80 (10.27)	40.29 (10.45)	0.028
MCS (Mental Component Score)	48.41 (8.28)	40.32 (12.26)	< 0.001
